# Use of radiographic and histologic scores to evaluate cats with idiopathic megacolon grouped based on the duration of their clinical signs

**DOI:** 10.3389/fvets.2022.1033090

**Published:** 2022-12-16

**Authors:** Ahmed Abdelbaset-Ismail, Nehal Ibrahim, Mohammed S. Sobh, Ahmed Ezzat Ahmed, Fatimah A. Al-Saeed, Amin A. Al-Doaiss, Khalid M. Al Syaad, Abd-Elmegeed Elmezyen, Mahmoud Abd-Elmaboud

**Affiliations:** ^1^Department of Surgery, Anesthesiology and Radiology, Faculty of Veterinary Medicine, Zagazig University, Zagazig, Egypt; ^2^Department of Pathology, Faculty of Veterinary Medicine, Zagazig University, Zagazig, Egypt; ^3^Department of Biology, College of Science, King Khalid University, Abha, Saudi Arabia; ^4^Department of Theriogenology, Faculty of Veterinary Medicine, South Valley University, Qena, Egypt; ^5^Department of Anatomy and Histology, Faculty of Medicine, Sana'a University, Sana'a, Yemen

**Keywords:** histopathology, cats, idiopathic megacolon, radiography, signs duration

## Abstract

Since the duration of clinical signs could be used to identify cases of chronic constipation, in addition, prolonged duration is often associated with irreversible changes. Thus, the main objective of this study was to determine whether the duration of clinical signs of idiopathic megacolon in cats affected their diagnosis and prognosis after treatment. Medical records of cats that either had confirmed megacolon for an unknown cause (cat patients) or with normal bowels (control cats) were reviewed. Cat patients were grouped based on the duration of their clinical signs (constipation/obstipation) to cats <6 months and ≥6 months. For all feline patients, abdominal radiographs (for colonic indexes) and resected colon specimens (for histology) were assessed vs. control cats. Treatment applied to cat patients was also evaluated. Cat patients were older (*p* = 0.0138) and had a higher maximum colon diameter (MCD; mean 41.25 vs. 21.67 mm, *p* < 0.0001) and MCD/L5L ratio (1.77 vs. 0.98, *p* < 0.0001) than controls. Compared to cats with <6 months, cats ≥6 months showed a higher MCD (43.78 vs. 37.12 mm, *p* < 0.0001) and MCD/L5L ratio (1.98 vs. 1.67, *p* < 0.0001). Histologically, increased thickness of the smooth muscularis mucosa (54.1 vs. 22.33 μm, *p* < 0.05), and inner circular (743.65 vs. 482.67 μm, *p* < 0.05) and outer longitudinal (570.68 vs. 330.33 μm, *p* < 0.05) smooth muscular layers of the muscularis externa was noted only in cat patients with ≥6 months compared to controls. Similarly, fewer ganglion cells (0.93 vs. 2.87, *p* < 0.005) and more necrotized myocytes (2.25 vs. 0.07, *p* < 0.005) were observed in cats with ≥6 months. In contrast to <6 months, the majority of cats (94.4%) with ≥6 months duration did not show any response to medical treatment and therefore underwent surgery with favorable results. In conclusion, this study suggests that the duration of clinical signs should be considered in conjunction with maximal colon scores to evaluate cats for idiopathic megacolon and determine the level of treatment. Functional abnormalities of the colonic smooth muscles may be a possible cause of idiopathic megacolon in cats.

## 1. Introduction

Megacolon is a descriptive term for an irretrievable, persistent colonic distension and occurs when the nerves and muscles of the colon are not functioning normally (1). Despite the term megacolon does not refer to information on underlying causes in cats, it is commonly a sequela of the advanced stage of prolonged untreatable constipation (2). Megacolon is commonly idiopathic and accounts for two-thirds of all other etiologies (3). Megacolon may occur secondary to acquired disorders such as neoplasia, foreign bodies, prostatic hyperplasia, perineal hernia, and neurogenic disorders, or congenital disorders such as atresia ani, atresia coli, and colorectal stricture (4). Megacolon can be readily diagnosed depending on a history of long-term constipation, clinical findings mainly distended colon on abdominal palpation, and plain abdominal radiography (5). On a radiograph, the colon diameter is calculated by a ratio of maximal colonic diameter (MCD) to the length of the body of the second (L2), fifth (L5), or seventh (L7) lumbar vertebra. It has been reported in cats that a ratio of MCD to L5 length is the most accurate measurement. In dogs and cats, the MCD should be lower than the length of the body of the L5 or L7 vertebra, and the MCD ratio of ≥1.5 in canines and ≥1.48 in felines is considered a strong marker for megacolon (6, 7). Management of megacolon is initially attempted by medical therapy to achieve easier evacuation of colonic hard contents (5). The common medications used include hyperosmotic or bulk-forming laxatives, enemas, and prokinetic drugs. Manual fecal extraction, as one of the conservative treatments for megacolon, could also be used along with general anesthesia (8). Since outcomes following medical therapy are often unsatisfactory, surgical intervention is the management of choice (9, 10). The mostly performed surgical management includes colotomy or subtotal/total colectomy with or without removal of ileocolic junction (ICJ) (10–13). Postoperatively, anorexia, inappetence, colotomy/colectomy incision dehiscence, abdominal incision dehiscence, septic peritonitis, and recurrent constipation are the common recorded complications (4). Since the duration of clinical signs could be used to identify cases of chronic constipation, in addition, prolonged duration is often associated with irreversible changes. Thus, the main purpose of this study was to address whether the duration of clinical signs of idiopathic megacolon is correlated with non-reversible colon changes in cats and simultaneously affects the diagnosis and prognosis post-treatment.

## 2. Materials and methods

The study protocol was designed and performed by the rules of the ethical committee and animal welfare of the Faculty of Veterinary Medicine, Zagazig University, Egypt (Protocol # ZU-IACUC/2/F/29/2022).

### 2.1. Data collection

Medical case records at Zagazig University Faculty of Veterinary Medicine were reviewed for the cats had megacolon confirmed between March 1, 2018, and March 31, 2021. Cats with confirmed idiopathic megacolon (of undetermined cause) were assessed. Cats with megacolon confirmed due to other concurrent disorders (e.g., perineal hernia, pelvic fracture malunion, foreign body, and perineal tumor) were excluded from the study. For the control cat group, abdominal radiographic images of cats were retrieved from medical records. These cats had no history or evidence of gastrointestinal disorders (based on case history and a negative fecal examination) or lumbar vertebral deformities either clinically or on radiographs. Cats with an MCD: L5L ratio <1.28 were considered clinically normal as previously reported (6) and thus included as control cats. According to a previous study (6), case cats with an MCD: L5L ratio >1.48 were categorized as megacolon and involved in this study, whereas values <1.48 were excluded from the study. Ultrasonography was not required for control and case cats to be included in this study.

For each cat, collected data included signalment, and history including duration of constipation/obstipation (tenesmus without defecation) prior to presentation. Radiographic and managemental data were also evaluated. Diagnosis of megacolon was initially done based on case history (mainly prolonged constipation), corresponding clinical findings, and clinical examination (e.g., abdominal palpation).

### 2.2. Radiographic examination

**Figure 1:**
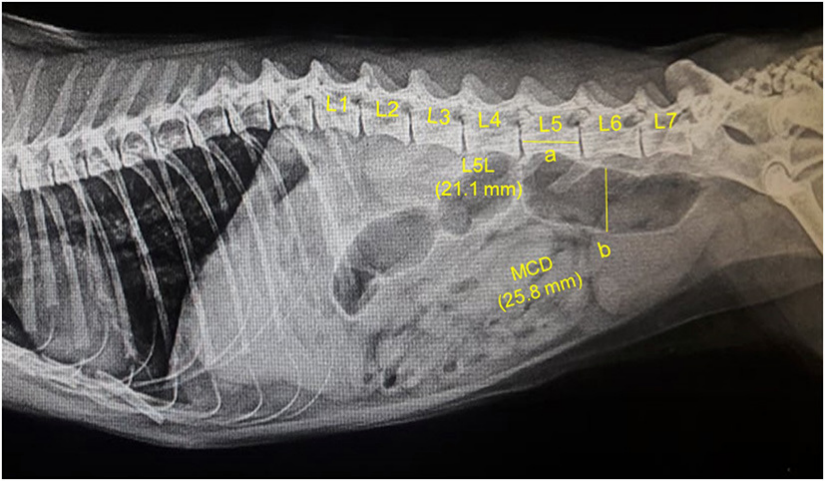
A representative lateral abdominal plain radiograph of a 4.5-years-old 5.7-kg Persian intact male normal healthy cat. Measurements for the body length of the fifth lumbar vertebra (L5L, labeled a, 21.1 mm), and maximum colon diameter (MCD, labeled b, 25.8 mm) are displayed by solid yellow lines.

**Figure 2:**
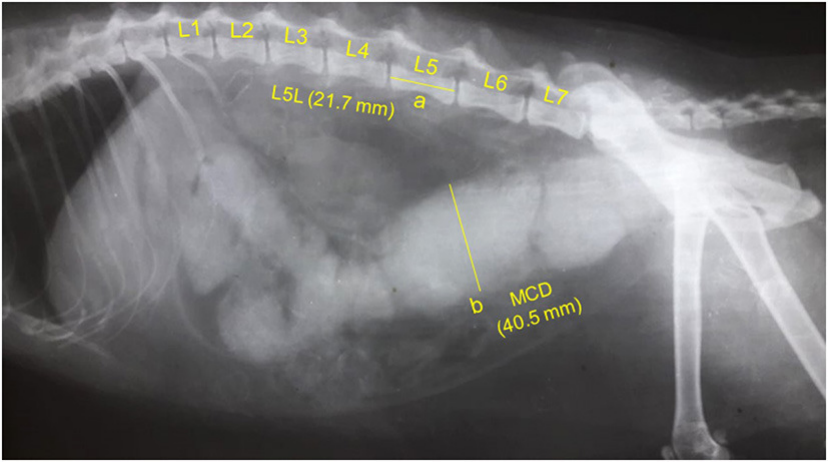
A representative lateral abdominal plain radiograph of a 6.9-years-old 4.6-kg domestic shorthair castrated male cat with idiopathic megacolon and constipation/obstipation. Measurements for the body length of the fifth lumbar vertebra (L5L, labeled a, 21.7 mm), and maximum colon diameter (MCD, labeled b, 40.5 mm) are displayed by solid yellow lines.

Abdominal radiographic images were obtained using a Toshiba Rotanode x-ray machine (POX-300BT, Japan) with standard exposure factors and object-to-focal distance. All abdominal radiographic images involved in the study were captured at the time of the inspiration-expiration pause of the imaged cat and without the use of sedative or anesthetic agents. At least, for each cat, a lateral abdominal radiograph was used to measure the MCD (mm), L5L, and MCD: L5L ratio as previously described (6). On a lateral abdominal radiograph, for all cats, MCD was measured at the most dilated point of the colon (Figures 1, 2), and the interfaces of the distended colon with either gas or feces were used to detect the serosal borders of the colon. At those points, the line was drawn perpendicular to the long axis of the colon as previously described (6). The length of vertebral L5 bodies was measured in millimeters. MCDs and L5Ls were assessed using image-processing software (Adobe© photoshop 8), on abdominal radiographic images adjusted at 200 dpi resolution and 100% scale size. After adjusting the resolution for all radiographic images obtained from cat patients, the MCD was measured at the most distended portion of the colon prior to the pelvic inlet where the line was drawn connecting the two serosal borders of the colon. All measurements were then made using a digital caliper. Ratios of the MCD to the length of L5 were then calculated for each cat.

### 2.3. Histopathological examination

Resection specimens were obtained from the colon of cat patients that had undergone surgery for megacolon. For this purpose, three cat patients had idiopathic megacolon of <6 months duration, and five had ≥6 months duration were used for histological analysis. Control colon specimens were obtained from five cats that had been euthanized for reasons other than an obstructive colon.

For both cat patients and controls, full-thickness colon resection specimens were collected, fixed in 10% paraformaldehyde, and then embedded in paraffin wax. Sections of 5 μm were obtained and processed for a standard hematoxylin and eosin staining as previously described (14). On the stained sections, the presence of histological features was noted. On the same stained sections, the thickness of the smooth muscularis mucosa and the two layers of smooth muscularis externa (inner circular and outer longitudinal muscular layers) was measured using Image J software (NIH, Bethesda, Maryland, USA). For each muscle, the mean of three measurements per section was calculated. The status of enteric myocytes was evaluated on a numeric score of 0–3 (normal to severely necrotized), and the number of ganglion cells was assessed also on a numeric score of 0–3 (absent to normal) in layers from serosal to the mucosal surface as previously described (15).

### 2.4. Cat patients grouping and management

Cases with idiopathic megacolon were grouped based on the duration of their clinical signs (constipation/obstipation) prior to presentation to cats with <6 months duration and cats with ≥6 months duration. Medical treatments applied to all cat patients included single or combination of enemas, stool softeners, manual de-obstipation, and 5-HT4 serotonergic agonist as a colon wall stimulant, Mosapride Citrate (Mosapride^®^, serotonin 5-TH4- receptor agonist, Western Pharmaceutical industries, Cairo, Egypt). Fluid therapy was administered to all cats. Low residues diets were prescribed to all patients. The non-responders to medical treatment were pre-operatively prepared and underwent subtotal colectomy (16).

### 2.5. Statistical analysis

Before running the analyses, we determined whether the data sets were normally distributed with D'Agostino-Pearson Test. Descriptive analysis was performed, and the obtained data were displayed as mean ± SD and with 95% confidence intervals (CIs) for the mean. An unpaired 2-tailed t-test was employed to compare means of MCDs, L5Ls, and MCD: L5L ratios across control vs. idiopathic megacolon cats, and case cats grouped by the duration of constipation/obstipation (idiopathic megacolon <6 months and idiopathic megacolon ≥6 months). Two-tailed Mann–Whitney *U*-test was used for comparing histological data across cat patients vs. controls. Data analysis was conducted with Prism GraphPad Software-version 7 (GraphPad Software Inc, California). For all analyses, statistically significant was set at a value of *p* < 0.05.

## 3. Results

### 3.1. Animals

The control group consisted of 30 healthy cats that were presented for reasons other than gastrointestinal disorders and had no detectable abnormalities on abdominal radiographic images. Among these cats, five were sexually intact females, eight were spayed females, seven were sexually intact males, and 10 were castrated males. Mixed breed cats [12/30 (40%)] were the most common presenting breeds (Table 1).

**Table 1:** Breed distributions of the cats that either was healthy without evidence of gastrointestinal disorders or had radiographically confirmed idiopathic megacolon and evaluated in this study.

**Breeds**	**No. (%) of control**	**No. (%) of cats**	
	**cats (*n* = 30)**	**with idiopathic megacolon**	
		**<6 months**	**≥6 months**
		**(12; 40%)**	**(18; 60%)**
Mixed breed	12 (40)	2 (6.7)	1 (3.3)
Domestic shorthair	7 (23.3)	6 (20)	9 (30)
Siamese	5 (16.7)	2 (6.7)	2 (6.7)
Domestic longhair	3 (13.3)	0	4 (16.7)
Persian	2 (6.7)	1 (3.3)	1 (3.3)
E. Mau	1 (3.3)	1 (3.3)	1 (3.3)

The cat patients contained 30 cats that had megacolon confirmed with abdominal radiography. Among these cats, six were females [sexually intact (4) and spayed (2)], and 24 were males [sexually intact (18) and castrated (6)]. Domestic shorthair cats [15/30 (50%)] were the most common presenting breed (Table 1). The mean duration of constipation/obstipation prior to presentation was 11.25 (SD: 5.66) months.

**Figure 3:**
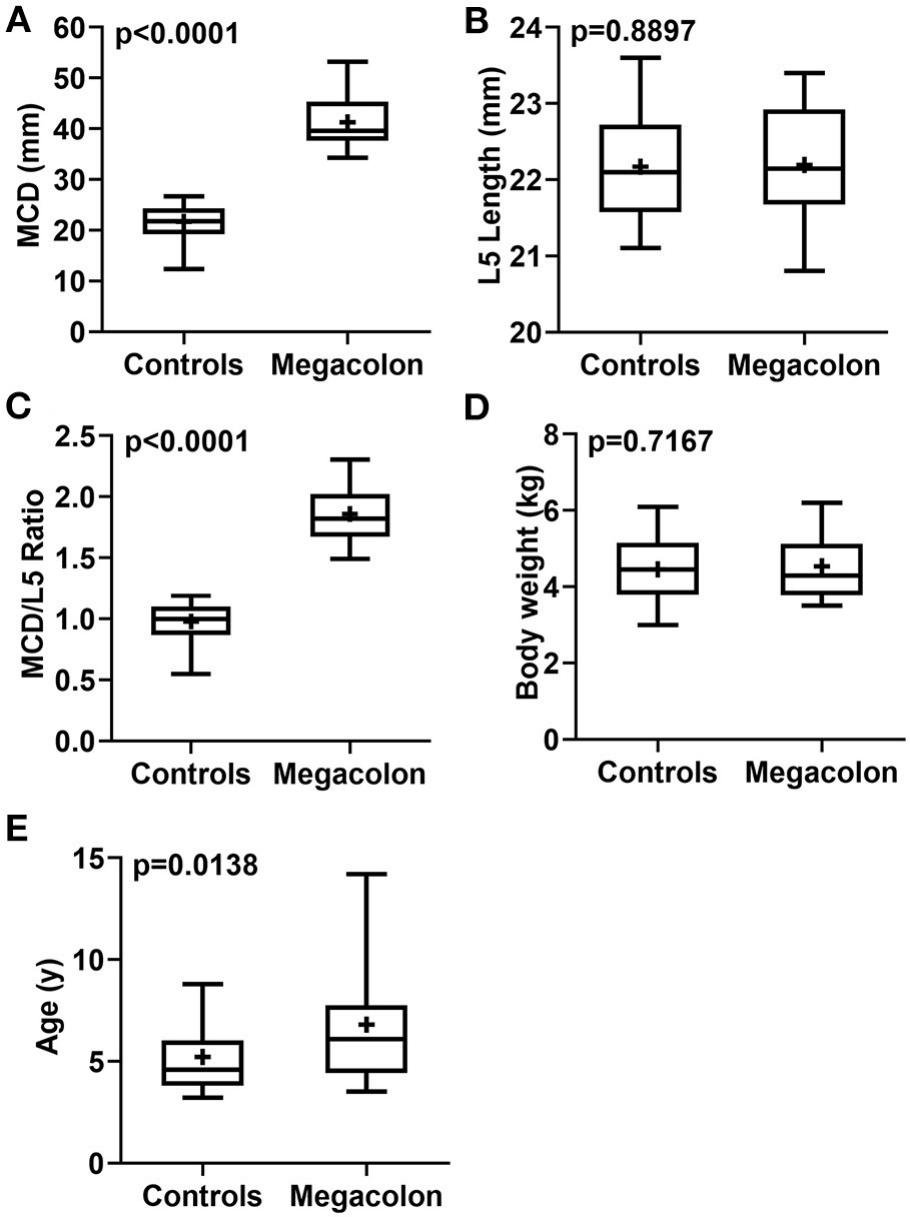
Box-and-whiskers plots of MCD **(A)**, L5L **(B)**, MCD/L5L ratio **(C)**, body weight **(D)**, and age **(E)** for cats that had confirmed idiopathic megacolon (*n* = 30) or healthy without evidence of either gastrointestinal or vertebral column diseases (controls; *n* = 30). The line represents the median, the cross represents the mean, and the whiskers rep-resents the range. Significant at *p* < 0.0001 and *p* < 0.005.

The mean ± SD age was significantly (*p* = 0.0138) higher in overall cats with idiopathic megacolon (6.81 ± 3.05 years), compared with control cats (5.21 ± 1.61 years). However, there was no obvious significant (*p* = 0.7167) difference in mean ± SD body weight for those overall cats had idiopathic megacolon (4.54 ± 0.87 kg) against control cats (4.46 ± 0.83 kg; Figure 3; Table 2).

**Table 2:** Descriptive analysis of MCD, L5L, MCD/L5L ratio, age, and body weight for the control cats (30) vs. the case cats (30) with idiopathic megacolon.

**Variables**	**Controls**		**Idiopathic megacolon**		***P*** **value**
	**Mean ±SD**	**95% CI (lower–upper)**	**Mean ±SD**	**95% CI (lower–upper)**	
MCD	21.67 ± 3.30	20.43–22.90	41.25 ± 5.02	39.38–43.12	< 0.0001
L5L	22.17 ± 0.72	21.90–22.44	22.20 ± 0.76	21.91–22.48	0.8897
MCD/L5L ratio	0.98 ± 0.15	0.92–1.03	1.86 ± 0.23	1.77–1.95	< 0.0001
Age (year)	5.21 ± 1.61	4.61–5.81	6.81 ± 3.05	5.67–7.95	0.0138
Body weight (kg)	4.46 ± 0.83	4.15–4.77	4.54 ± 0.87	4.21–4.86	0.7167

**Figure 4:**
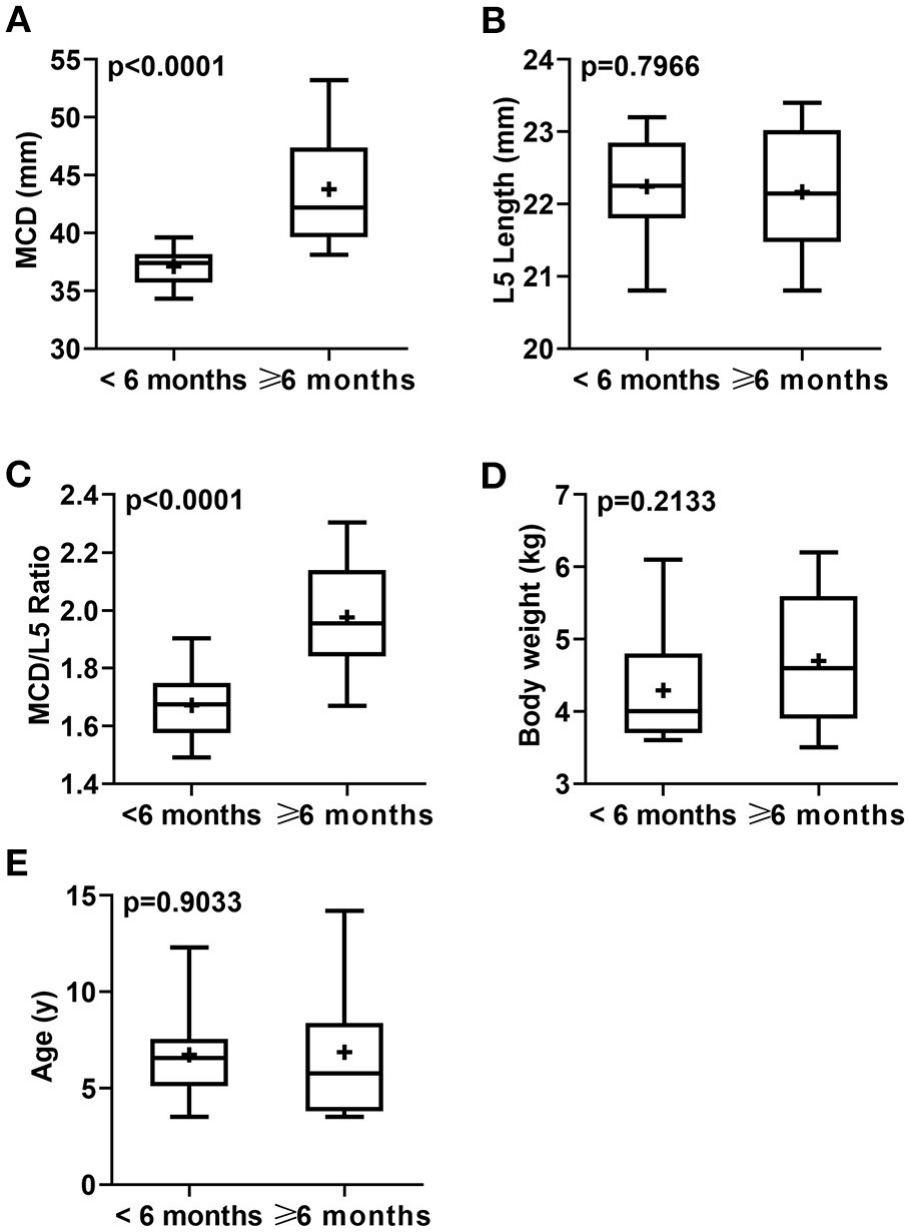
Box-and-whiskers plots of MCD **(A)**, L5L **(B)**, MCD/L5L Ratio **(C)**, body weight **(D)**, and age **(E)** for cats that had confirmed idiopathic megacolon with a duration of constipation/obstipation <6 months (*n* = 12) or ≥6 months (*n* = 18). The line represents the median, the cross represents the mean, and the whiskers represent the range. Significant at *p* < 0.0001 and *p* < 0.005.

According to the duration of constipation/obstipation, cat patients were sub-grouped into <6 months [12/30 (40%)], and ≥6 months [18/30 (60%)]. There were no considerable differences in mean age (*p* = 0.9033) and mean body weight (*p* = 0.2133) for idiopathic megacolon subgroups: <6 months (6.73 ± 2.42 years and 4.29 ± 0.79 kg, respectively) vs. ≥6 months (6.87 ± 3.47 years and 4.70 ± 0.90 kg, respectively; Figure 4; Table 3).

**Table 3:** Descriptive analysis of MCD, L5L, MCD/L5L ratio, age, and body weight for the cats had confirmed idiopathic megacolon with either duration of constipation/obstipation <6 months (*n* = 12) or ≥6 months (*n* = 18).

**Variables**	<**6 months idiopathic megacolon**		≥**6 months idiopathic megacolon**		***P*** **value**
	**Mean ±SD**	**95% CI (lower–upper)**	**Mean ±SD**	**95% CI (lower–upper)**	
MCD	37.12 ± 1.72	36.03–38.21	43.78 ± 4.69	41.45–46.12	< 0.0001
L5L	22.24 ± 0.69	21.80–22.69	22.17 ± 0.82	21.76–22.57	0.7966
MCD/L5L ratio	1.67 ± 0.11	1.60–1.74	1.98 ± 0.20	1.88–2.08	< 0.0001
Age (year)	6.73 ± 2.42	5.19–8.26	6.87 ± 3.47	5.14–8.59	0.9033
Body weight (kg)	4.29 ± 0.79	3.79–4.79	4.70 ± 0.90	4.25–5.15	0.2133

### 3.2. Radiographic findings

Measurements of MCD, L5L, and MCD:L4L ratio were collected and analyzed. Relative to the control cats, the mean ± SD MCD values were significantly (*p* < 0.0001) higher in overall cats with idiopathic megacolon (21.67 ± 3.30 vs. 41.25 ± 5.02), but as expected, means ± SD values of L5L did not differ significantly (*p* = 0.8897) for cats with idiopathic megacolon (22.20 ± 0.76) vs. control cats (22.17 ± 0.72). The mean ± SD value of the MCD:L4L ratio for cats with megacolon (1.86 ± 0.23) was significantly greater (*p* < 0.0001) than that in control cats (0.98 ± 0.15; Figure 3; Table 2).

Among megacolon cases grouped by the duration of constipation/obstipation, there were significant differences between <6 months, and ≥6 months subgroups in means ± SD MCD (*p* < 0.0001; 37.12 ± 1.72 and 43.78 ± 4.69, respectively), and means ± SD values of MCD:L5L ratio (*p* < 0.0001; 1.67 ± 0.11 and 1.98 ± 0.20, respectively). There was no detectable significant variation of L5L between these two subgroups (*p* = 0.7966, 22.24 ± 0.69, and 22.17 ± 0.82, respectively; Figure 4; Table 3).

None of these overall cats included in this study had radiographic evidence of lumbar vertebral deformities.

### 3.3. Histopathological findings

Resection specimens were obtained from the colon of eight cat patients (6 males, mean ± SD age 7.8 ± 3.13 years, and two females, mean ± SD age 4.25 ± 0.92 years) that had undergone surgery for megacolon.

Control colon specimens were obtained from five cats (three males, mean ± SD age 3.85 ± 1.02 years, and two females, mean ± SD age 3.45 ± 0.07 years) which had been euthanized for reasons other than an obstructive colon. None of these cats had notable colonic abnormalities.

The histological analyses of the resected colon specimens were performed to investigate why idiopathic megacolon of ≥6 months duration had a significant impact on responding to medical treatment and ultimately necessitated surgical intervention. Histological analyses of the resected colon specimens were performed. Of note, we could not analyze for cats that had <6 months duration and responded to medical treatment, however, three cats of this subgroup that failed to respond to medication and underwent surgery were included. Normal colon samples were collected from cats that had been euthanized for reasons rather than gastrointestinal disorders.

#### 3.3.1. Descending colon

**Figure 5:**
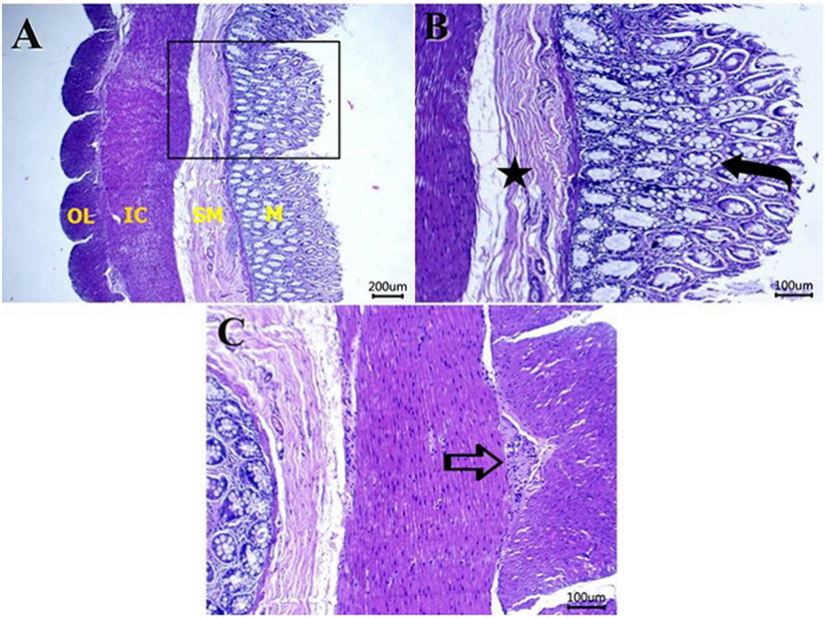
Photomicrograph of H&E colon sections from healthy cat colon showing: **(A)** normal histology of mucosa (M), submucosa (SM), inner circular (IC), and outer longitudinal (OL) smooth muscles. Scale bar 200 μm. **(B)** Numerous goblet cells within the lamina epithelialis (curved arrow), and no morphological changes in the submucosal layer (star label). Scale bar 100 μm. **(C)** Normal myenteric plexus (arrow) between the inner and outer muscular layers. Scale bar 100 μm.

**Figure 6:**
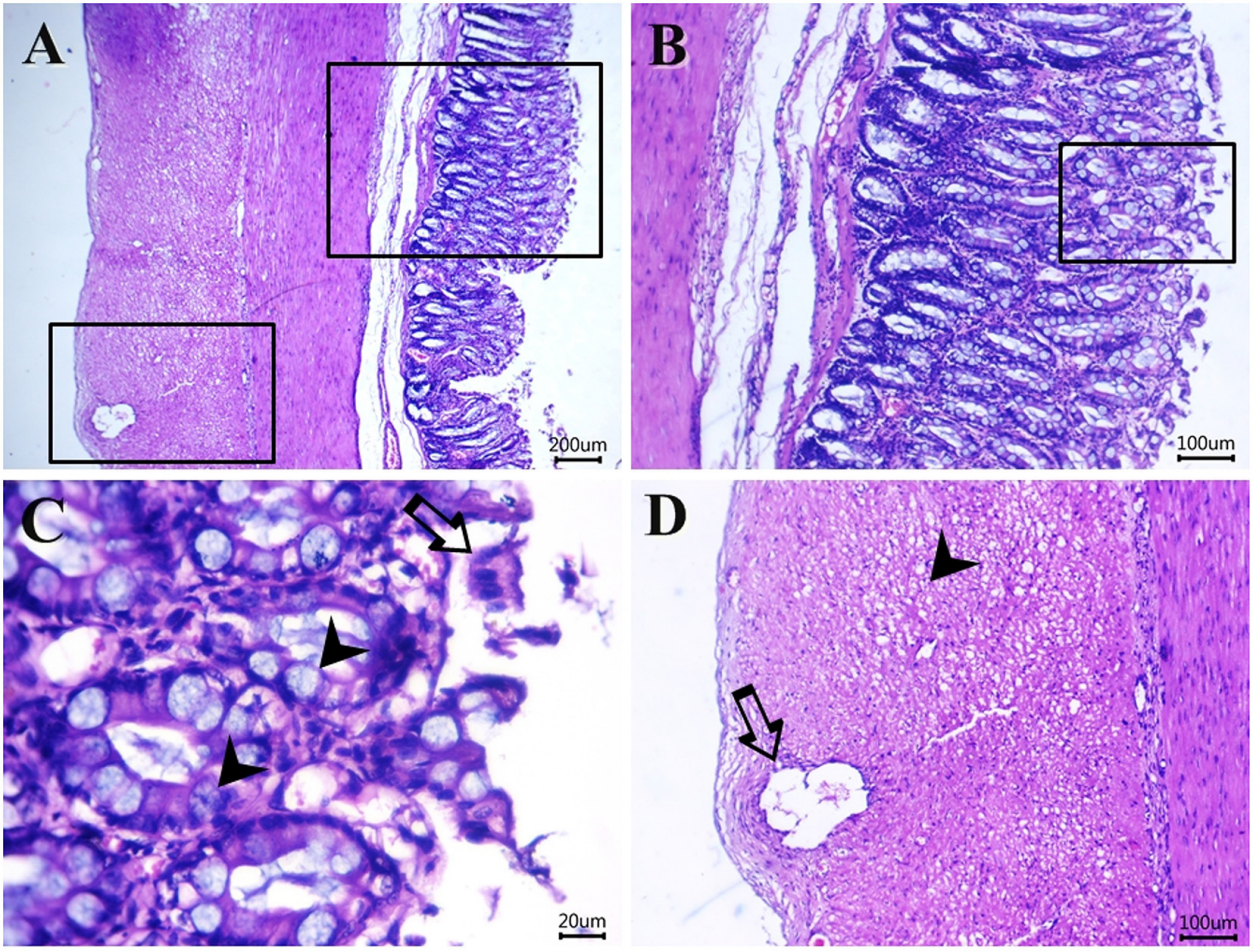
Photomicrograph of H&E-stained sections from cat idiopathic megacolon that had <6 months duration of constipation/obstipation showing mild-moderate degenerative changes: **(A–C)** desquamated epithelium (arrow) and metaplastic changes of the colonic columnar epithelium into goblet cells (arrowheads). **(D)** Vacuolated (arrowhead) and necrotic enteric myocytes (arrow). Scale bar 200, 100, 20, and 100 μm, respectively.

**Figure 7:**
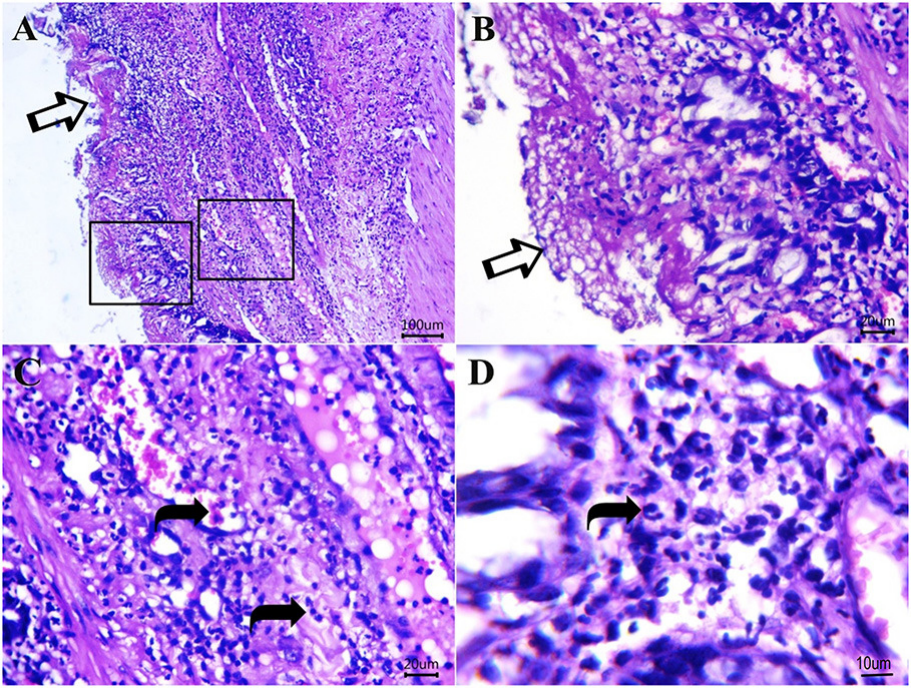
Photomicrograph of H&E sections from cat idiopathic megacolon that had ≥6 months duration of constipation/obstipation showing moderate to severe degenerative changes. **(A, B)** The diphtheritic membrane is replaced by most mucosal layers (arrows). Scale bar 100 and 20 μm, respectively. **(C, D)** Neutrophils (curved arrows) and proteinaceous exudates within lamina propria and submucosa. Scale bar 20 and 10 μm, respectively.

**Figure 8:**
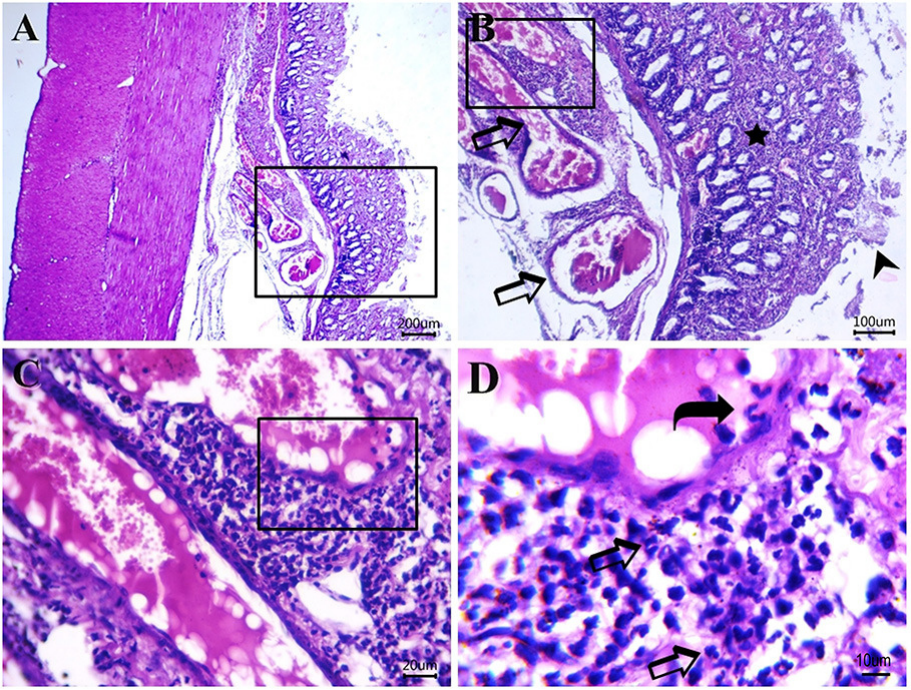
Photomicrograph of H&E-stained sections from cat idiopathic megacolon that had ≥6 months duration of constipation/obstipation showing moderate to severe degenerative changes: **(A, B)** Catarrhal lesions in the colonic epithelium [desquamated colonic epithelium (arrowhead), inflammatory cells infiltrate (star) between degenerated and necrotized colonic glands and congested blood vessels (arrow)]. **(C, D)** Submucosal neutrophils infiltrations (arrows) and clearly visible adhered neutrophils to blood vessel endothelium (curved arrow) for the transmigration process. Scale bar 200, 100, 20, and 10 μm, respectively.

Figures 5–8 show the detected histological features in colon sections obtained from normal cats (Figure 5), cats with idiopathic megacolon <6 months duration (Figure 6), and cats with idiopathic megacolon ≥6 months duration (Figures 7, 8).

**Figure 9:**
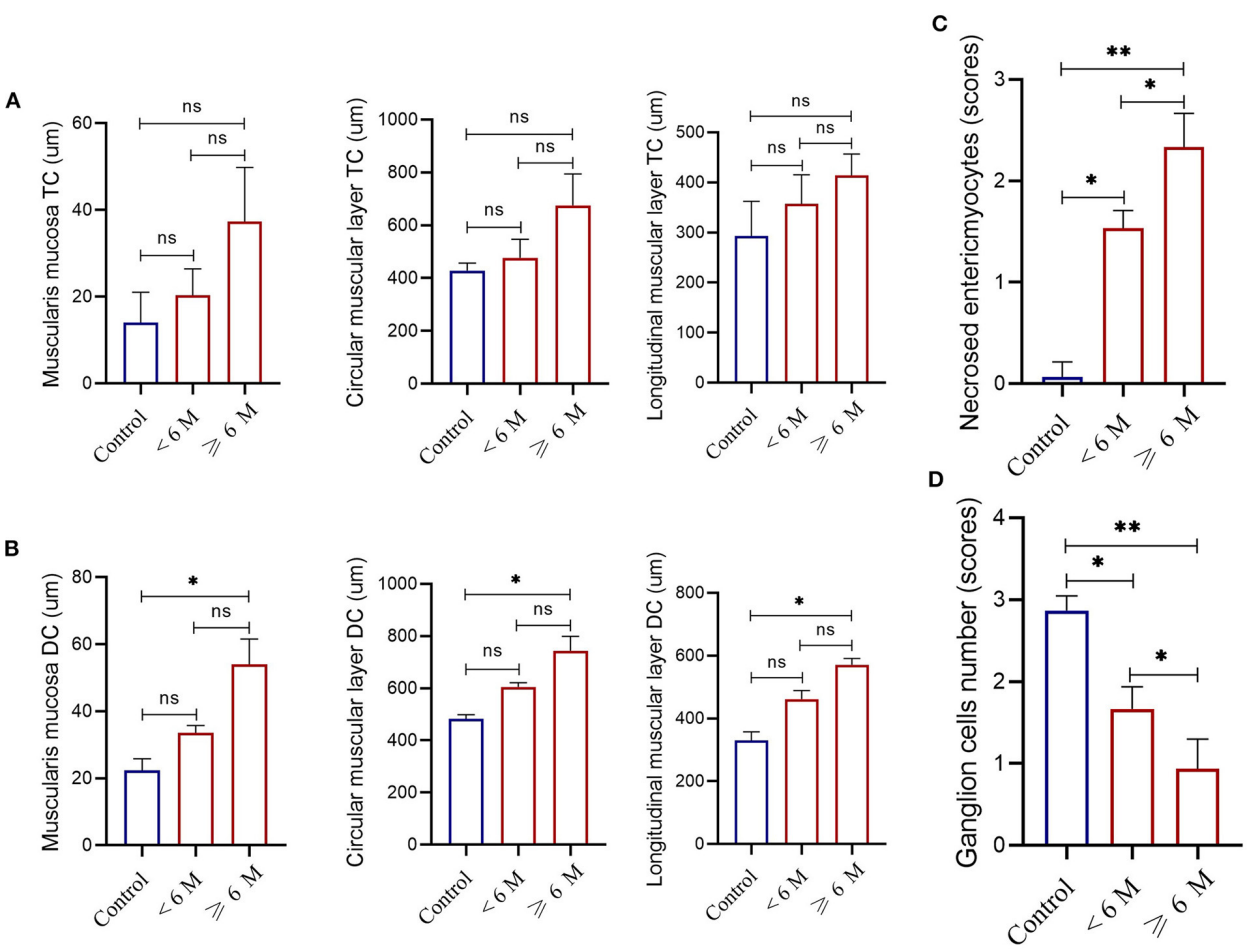
Measurements of the thickness of the muscularis mucosa, and the inner circular and outer longitudinal smooth muscle layers of the muscularis externa obtained from the transverse colon [TC, **(A)**] or descending colon [DC, **(B)**] in controls (*n* = 5) and case cats with idiopathic megacolon and grouped based on the duration of constipation/obstipation, <6 months (*n* = 3) and ≥6 months (*n* = 5). Necrotized enteric myocytes **(C)** and ganglion cells **(D)** mean scores in controls and grouped case cats with idiopathic megacolon. Data are given as mean ± SD. ns, not significant. *p < 0.05; **p < 0.005.

For cat patients with idiopathic megacolon <6 months duration, there was no thickening in the muscularis mucosa (means ± SD 33.67 ± 2.08 μm vs. control means ± SD 22.33 ± 3.51 μm), and inner circular muscular layer (means ± SD 605.67 ± 15.7 μm vs. control means ± SD 482.67 ± 16.17 μm) and outer longitudinal muscular layer (means ± SD 461.33 ± 27.68 μm vs. control means ± SD 330.33 ± 27.15 μm) of smooth muscularis externa. However, there was a significant increase in the mean ± SD necrosed myocytes (means ± SD 1.33 ± 0.33) and a considerable reduction of the myenteric ganglion cells (means ± SD 1.78 ± 0.19) relative to controls (means ± SD 0.067 ± 0.15 and 2.87 ± 0.18, respectively; Figure 9).

For cat patients with idiopathic megacolon of ≥6 months duration, there was notable thickening in the smooth muscularis mucosa (mean ± SD 54.1 ± 7.55 μm vs. control 22.33 ± 3.51 μm), and in both two layers of the smooth muscularis externa, inner circular muscular layer (means ± SD 743.65 ± 55.01 μm vs. control means ± SD 482.67 ± 16.17 μm) and outer longitudinal muscular layer (means ± SD 570.68 ± 20. 56 μm vs. control means ± SD 330.33 ± 27.15 μm). Similarly, we observed a significant increase for mean ± SD necrosed myocytes (2.25 ± 0.32 vs. control 0.067 ± 0.15) and a significant reduction of ganglion cells (0.93 ± 0.38 vs. control 2.87 ± 0.18; Figure 9).

#### 3.3.2. Transverse colon

None of the three smooth muscle layers at the transverse colon of all cats with idiopathic megacolon showed abnormal changes, compared with normal bowels (Figure 9A). For muscularis mucosa: [<6 months: (*p* = 0.3; 20.33 ± 6.11 μm vs. control 14.0 ± 7.0 μm)] and [≥6 months: (*p* = 0.07; 37.33 ± 12.50 μm vs. control 14.0 ± 7.0 μm)], for inner circular muscular layer: [<6 months: (*p* = 0.35; 477.0 ± 69.94 μm vs. control 428.33 ± 28.50 μm)] and [≥6 months: (*p* = 0.07; 675.67 ± 119.22 μm vs. control 428.33 ± 28.50 μm)], and for outer longitudinal muscular layer: [<6 months: (*p* = 0.28; 357.33 ± 57.71 μm vs. control 293.00 ± 69.35 μm)] and [≥6 months: (*p* = 0.08; 414.00 ± 43.01 μm vs. control 293.00 ± 69.35 μm)].

### 3.4. Treatment and outcome

Cats with idiopathic megacolon and grouped based on their duration of clinical constipation/obstipation to <6 months (12/30, 40%) and ≥6 months (18/30, 60%) subgroups were assessed for the treatment outcomes. Medical treatment was initially applied to all cat patients. Table 4 summarizes the medical treatment offered to cat patients. In the case of <6 months cats, medical treatment was satisfactory in 8/12 (66.67%) and was unsatisfactory in 4 (33.33%). One (8.33%) of these cats died and the remaining three (25%) cats had re-current constipation/obstipation. In the case of ≥6 months cats, medical treatment was favorable in only 1/18 (5.56%) cats and was unsatisfactory in 17/18 (94.44%) cases. Of the latter, two (11.11%) cats died, and 15 (83.33%) had unresolved symptoms of constipation/obstipation. Eighteen cats that did not respond to medical treatment had eventually undergone surgery “subtotal colectomy.” Thirteen (72.22%) of these cases showed favorable outcomes in which the symptoms resolved, and five (27.78%) cats had unfavorable outcomes. Of these five cats, one died, one was euthanized, and the remaining three continued to experience signs of constipation/obstipation post-surgery and their follow-up was lost.

**Table 4:** Conservative treatment offered to the cat patients diagnosed with idiopathic megacolon and grouped into two subgroups based on the duration of clinical signs.

**Treatment**	**Dosage**	**Idiopathic megacolon**	
		**<6 months**	**≥6 months**
		**(*n* = 12)**	**(*n* = 18)**
Fluid therapy	100–150 ml/cat (S/C or IV, 3 ml/kg/h)	12	18
Enemas	Warm water (50 ml) + mineral oil (20 ml)	2	3
Stool softeners	Lactulose (0.5 ml/kg, orally, twice a day) Mineral oil (5 ml/cat, orally, twice daily)	1	1
Enemas + stool softeners		5	6
Enemas + stool softeners + manual de-obstipation		4	8
Mosapride citrate	5 mg/cat (twice a day)	12	18

## 4. Discussion

The salient finding in this study is that prolonged duration of clinical signs of idiopathic megacolon in cats ≥6 months was associated with irreversible functional changes in the colon. Hence, the existence of these non-reversible changes was accompanied by ineffective medical treatment, and accordingly, subtotal colectomy was performed with applauding results. Moreover, we found based on radiographic and clinical data of cats with idiopathic megacolon a clear association between the degree of colonic dilation and diagnosis, treatment strategy, and consequently prognosis.

Megacolon is irreversible dilation and dysmotility of the colon and eventually resulted from chronic constipation/obstipation. In felines, idiopathic megacolon has been recently reported to affect an estimated 182 cats during 18 years within 18 veterinary hospitals worldwide (17). Improving the prognosis of megacolon is challenging which is in part due to the difficulty in treating cats with megacolon medically. Despite subtotal/total colectomy being the treatment of choice (18), however, its outcomes are still challenging (3). Thus, identification and treatment outcomes of idiopathic megacolon within cats residing in Egypt are in demand not only for pet clinicians but also for feline owners. Also, it could be challenging to evaluate the megacolon of cats in emergencies. In this study, the most presenting cats with megacolon were domestic shorthair breeds, which is comparable to another study where 61.4% of cats were domestic shorthairs (17). We also found male cats were presented with 6.1 years (SD: 3.05) median age and 4.3 kg (SD: 0.87) weight, relative to another study where they reported the median age and weight of the affected cats were 8.6 years and 5.3 kg, respectively (17).

In the present study, cats with idiopathic megacolon had obstipation (tenesmus without defecation), which is similarly presented by another study, as the principal clinical sign (17). Obstipation means there is no bowel motility, whereas constipation means there are about ≤ 3 bowel motility per week (5). Dysfunction of colon motility leads to the retention of voluminous fecal material along the colon that is most revealing in painful abdominal palpation and digital rectal examination, as reported in this study. The duration of clinical signs is the primary difference between obstipation and constipation. In this study, the median duration of clinical signs before admittance was 11.25 months (SD: 5.66). Another study demonstrated that the median preoperative duration of clinical signs of megacolon in cats was 24 months (IQR, 12–42 months) (17).

More importantly, herein, in feline cases having ≥6 months duration of clinical signs showed unsatisfactory medical treatment due to irreversible colonic changes, hence sub-total colectomy was the only intervention of choice with satisfactory results. In this study, an abdominal radiograph survey was potentially sufficient to confirm a diagnosis of idiopathic megacolon in the studied cases (6). We found that normal colon diameter and MCD/L5 length ratio were 21.3 mm (SD: 4.3), and 0.96 mm (SD: 0.2; range: 0.8–1.15), respectively, whereas cats with megacolon had 39.6 mm (SD: 5.03; range: 34.3–53.2) and 1.82 mm (SD: 0.23; range: 1.51–2.3), respectively. Comparably with other studies where normal MCD was 21.2 ± 2.5 mm and MCD/L5 ratio was <1.28, and in megacolon, cases were 36.9 ± 7.2 mm and >1.48, respectively (6). Herein, we calculated a ratio of MCD to L5 body length since it is considered the most repeatable and accurate measurement compared to the ratio of MCD to L2 or L7 (6). In some cats with constipation, MCD was ≤ 27 mm, which is considered another difference between constipation and megacolon on the radiograph (6). In dogs with colonic impaction, the ratio of MCD to L2 was 1.9 (1.3–3.4) and the ratio of MCD to L5 was 1.7 (1.2–3.1). The authors of this study reported that these ratios were not associated with the suggested idiopathic or secondary megacolon (8).

Medical approaches as an initial trial clinically differ according to the degree of constipation/obstipation. Enemas, stool softeners, manual removal of fecal material, and bowel-motility stimulants (pro-kinetics) could be used singly or in combination for the treatment of mild or moderate degrees of constipation (4). In addition to these de-obstipation medications, fluid therapy is required in severe degrees of constipation/ obstipation for ameliorating water, acid-base, and electrolyte imbalances (8). The cases of obstipation which did not respond to the medical treatment were found associated most likely with megacolon of duration ≥6 months, in which the subtotal colectomy was the definitive treatment. It has been reported that the medical approach was highly successful in dogs suffering chronic impaction. Additionally, secondary megacolon in 9 dogs was effectively treated with subtotal colectomy (8). Moreover, in cats, a retrospective study has shown that sub-total colectomy was highly successful in treating idiopathic megacolon with an improvement in survival rate, however, excision of ICJ was accompanied by less satisfying outcomes (17).

In this study, the majority of cases having <6 months duration of clinical signs showed successful results after de-obstipation medications were applied, whereas the cases having ≥6 months duration underwent subtotal colectomy and consequently a favorable prognosis was obtained 10 days post-operatively in the majority of them. The reported mortality rate of cats who underwent subtotal colectomy and/or ICJ removal was 5.6% and on long-term follow-up, 14% of cats died/were euthanized (17). Herein, the long-term follow-up demonstrated 20% (6/30) of cases died or had been euthanized owing to health deterioration for unknown reasons.

Although histological abnormalities in human megacolon have been well-defined, the etiopathogenesis of idiopathic megacolon in cats is unknown. More specifically, it is not known whether abnormalities involving colonic intrinsic innervation, smooth muscles, or both, lead to colonic dilation and dysmotility. As mentioned earlier, the pathology of idiopathic megacolon was variable as the smooth muscles of the colon appeared normal (19), hypertrophic (20), or atrophic (21). Moreover, the nerve plexus of idiopathic megacolon has been shown to be normal (21) or associated with a decrease in the number of ganglion cells (22). In this study, we found that cats with idiopathic megacolon of ≥6 months duration showed thickening in the smooth muscle layers of the descending colon. However, in cat patients with <6 months duration and had undergone surgery after failure of medical treatment, we found no thickening was observed in smooth muscle layers of both the transverse and descending colon. More interestingly, thickened smooth layers were associated with a decrease in the number of colonic ganglion cells and an increase in the number of enteric myocytes indicating a change in intrinsic innervation and colon motility. Taken together, these colon abnormalities may explain why idiopathic megacolon of ≥6 months is associated with unfavorable outcomes after medical treatment.

## 5. Conclusions

We show that cats with idiopathic megacolon ≥6 months duration had (1) an average ratio of MCD/L5L 1.98 ± 0.20, (2) an increased thickness of colonic smooth muscle layers, (3) a decrease in the number of ganglion cells, and (4) an increase in the number of necrotized enteric myocytes. All these observations could be suggested as possible underlying causes of non-reversible functional colonic changes, thus surgical intervention is required rather than medical treatment.

## Data availability statement

The original contributions presented in the study are included in the article/supplementary material, further inquiries can be directed to the corresponding author/s.

## Ethics statement

The animal study was reviewed and approved by Ethical Committee and Animal Welfare of the Faculty of Veterinary Medicine, Zagazig University, Egypt (Protocol # ZU-IACUC/2/F/29/2022). Written informed consent was obtained from the owners for the participation of their animals in this study.

## Author contributions

Conceptualization, formal analysis, and investigation: AA-I, MA-E, and A-EE. Methodology and writing—original draft preparation: AA-I, NI, AA-D, and MS. Software: AA-I, AA, and FA-S. Validation and supervision: MA-E and A-EE. Data curation: KA. Writing—review and editing: MA-E, KA, and A-EE. Visualization: NI and MS. Project administration: MA-E, FA-S, AA-D, and A-EE. Funding acquisition: FA-S, AA-D, and KA. All authors have read and agreed to the published version of the manuscript.

## References

[1] Washabau RJ Holt D. Pathogenesis, diagnosis, and therapy of feline idiopathic megacolon. Vet Clin North Am Small Anim Pract. (1999) 29:589–603. 10.1016/S0195-5616(99)50036-3 10202804

[2] Garcia-Pertierra S Gonzalez-Gasch E Catala Puyol C Closa Boixeda JM. Dynamic chronic rectal obstruction causing a severe colonic dilatation in a cat. JFMS Open Rep. (2017) 3:2055116917725222. 10.1177/2055116917725222 28839947 PMC5565026

[3] Rosin E Walshaw R Mehlhaff C Matthiesen D Orsher R Kusba J. Subtotal colectomy for treatment of chronic constipation associated with idiopathic megacolon in cats: 38 cases (1979-1985). J Am Vet Med Assoc. (1988) 193:850–3. 3192467

[4] Williams JM. Colon. In:Tobias KM Johnston SA, editors. Veterinary Surgery: Small Animal. Saint Louis, MO: Elsevier/Saunders (2012), p. 1542–61.

[5] Bertoy RW. Megacolon in the cat. Vet Clin North Am Small Anim Pract. (2002) 32:901–15. 10.1016/S0195-5616(02)00020-7 12148317

[6] Trevail T Gunn-Moore D Carrera I Courcier E Sullivan M. Radiographic diameter of the colon in normal and constipated cats and in cats with megacolon. Vet Radiol Ultrasound. (2011) 52:516–20. 10.1111/j.1740-8261.2011.01830.x 21599794

[7] Lee R Leowijuk C. Normal parameters in abdominal radiology of the dog and cat. J Small Anim Pract. (1982) 23:251–69. 10.1111/j.1748-5827.1982.tb01664.x

[8] Tzimtzimis E Papazoglou L Patsikas M Tsioli V Kouti V Konstantinidis A. Colonic impaction in dogs: a retrospective study of 58 cases (1996 to 2014). J Small Anim Pract. (2019) 60:444–9. 10.1111/jsap.13007 31025712

[9] Gregory CR Guilford WG Berry CR Olsen J Pederson NC. Enteric function in cats after subtotal colectomy for treatment of megacolon. Vet Surg. (1990) 19:216–20. 10.1111/j.1532-950X.1990.tb01173.x 2349778

[10] Barnes DC. Subtotal colectomy by rectal pull-through for treatment of idiopathic megacolon in 2 cats. Can Vet J. (2012) 53:780−2. 23277646 PMC3377462

[11] Ryan S Seim H 3rd Macphail C Bright R Monnet E. Comparison of biofragmentable anastomosis ring and sutured anastomoses for subtotal colectomy in cats with idiopathic megacolon. Vet Surg. (2006) 35:740–8. 10.1111/j.1532-950X.2006.00218.x 17187636

[12] Kudisch M Pavletic MM. Subtotal colectomy with surgical stapling instruments *via* a trans-cecal approach for treatment of acquired megacolon in cats. Vet Surg. (1993) 22:457–63. 10.1111/j.1532-950X.1993.tb00422.x 8116201

[13] Bright RM. Idiopathic megacolon in the cat: subtotal colectomy with preservation of the ileocolic valve. Vet Med Rep. (1991) 183–7.

[14] Suvarna KS Layton C Bancroft JD. Bancroft's Theory and Practice of Histological Techniques, 8th ed. Amsterdam: Elsevier Ltd. (2019).

[15] Gibson-Corley KN Olivier AK Meyerholz DK. Principles for valid histopathologic scoring in research. Vet Pathol. (2013) 50:1007–15. 10.1177/0300985813485099 23558974 PMC3795863

[16] Bright RM Burrows CF Goring R Fox S Tilmant L. Subtotal colectomy for treatment of acquired megacolon in the dog and cat. J Am Vet Med Assoc. (1986) 188:1412–6. 3744968

[17] Grossman RM Sumner JP Lopez DJ Dornbusch JA Singh A Lux CN. Evaluation of outcomes following subtotal colectomy for the treatment of idiopathic megacolon in cats. J Am Vet Med Assoc. (2021) 259:1292–9. 10.2460/javma.20.07.0418 34727062

[18] Rosin E. Megacolon in cats. The role of colectomy. Vet Clin North Am Small Anim Pract. (1993) 23:587–94. 10.1016/S0195-5616(93)50307-8 8503161

[19] Stabile G Kamm MA Phillips RK Hawley PR Lennard-Jones JE. Partial colectomy and coloanal anastomosis for idiopathic megarectum and megacolon. Dis Colon Rectum. (1992) 35:158–62. 10.1007/BF02050671 1735317

[20] Stabile G Kamm MA Hawley PR Lennard-Jones JE. Results of the Duhamel operation in the treatment of idiopathic megarectum and megacolon. Br J Surg. (1991) 78:661–3. 10.1002/bjs.1800780609 2070228

[21] Gattuso JM Kamm MA Talbot JC. Pathology of idiopathic megarectum and megacolon. Gut. (1997) 41:252–7. 10.1136/gut.41.2.252 9301507 PMC1891468

[22] Krishnamurthy S Schuffler MD Rohrmann CA Pope CE 2nd. Severe idiopathic constipation is associated with a distinctive abnormality of the colonic myenteric plexus. Gastroenterology. (1985) 88:26–34. 10.1016/S0016-5085(85)80128-1 3964770

